# Single course of high dose dexamethasone is more effective than conventional prednisolone therapy in the treatment of primary newly diagnosed immune thrombocytopenia

**DOI:** 10.1186/2008-2231-20-7

**Published:** 2012-08-28

**Authors:** Mohammad Ali Mashhadi, Mahmoud Ali Kaykhaei, Zahra Sepehri, Ebrahim Miri-Moghaddam

**Affiliations:** 1 Genetics research center, Division of Oncology and Hematology, Department of Medicine, Zahedan University of Medical Sciences, Zahedan, Iran; 2 Zahra Sepehri, Department of internal medicine, Zabol University of Medical Sciences, Zabol, Iran; 3 Genetics of Non-Communicable Disease Research Center, Zahedan University of Medical Sciences, Zahedan, Iran

**Keywords:** High dose dexamethasone, Immune thrombocytopenia, Prednisolone

## Abstract

**Introduction:**

Immune thrombocytopenia (ITP) is an immune disorder commonly presents as isolated thrombocytopenia. Generally corticosteroids are the main treatment of ITP. This study was designed to evaluate effectiveness of high dose dexamethasone comparing conventional corticosteroid therapy in the treatment of ITP.

**Materials and methods:**

In a randomized prospective study, sixty adult patients with newly diagnosed primary symptomatic ITP (Platelet count < 20,000) were evaluated. Patients divided into two groups. In group A, thirty patients (mean age of 24.9 years) received Dexamethasone 40 mg/IV/daily for four days (10 mg/q6h); and then Prednisolone 1 ^mg^/_kg_/day/PO with rapid tapering of prednisolone (10 mg/week). From the other hand, in group B, thirty patients (mean age of 27.2 years) were treated with Prednisolone 1 ^mg^/_kg_/day/PO for four weeks, then the drug tapered weekly.

**Results:**

All the patients in group A showed favorable response within the first seven days, 27 cases presented complete response (CR) and three cases revealed response (R). In group B, 11 cases had CR, 13 cases showed R and six cases had No response (NR). After three months, rates of CR were 80% and 23.3% in group A and B; respectively. Responses were 16.7% and 33.3%, NRs were 6.6% and 43.3% in group A and B; respectively (P < 0.0001). After 6 months, CR was 73.3% vs.16.7%, and R was 16.7% vs.36.7% and NR was 10% vs. 46.7% in group A and B; respectively (P < 0.0001). After 12 months, there was no change in response rate in group A, but in group B 53% were non responsive, 40% showed R (chronic ITP) and complete response was observed only in 6.7% (P < 0.0001). Three cases in group A and 12 cases in group B had needed splenectomy (P < 0.00002).

**Conclusion:**

We showed that high dose dexamethasone is more effective than conventional steroid therapy in newly diagnosed ITP as initial treatment with less relapses and toxicities.

## Introduction

The primary immune thrombocytopenia (ITP) is an isolated thrombocytopenia without exogenous etiologic factors and secondary causes ( [1,2]). Pathogenesis of ITP is due to platelet specific autoantibodies (most commonly IgG type) that bind to the platelet surface and cause clearance of them in spleen and liver ( [3,4]). Treatment in patients with ITP is dynamic and may alter in various situations. Patients with life threatening bleeding may need treatment with glucocorticoids, IVIG, platelet transfusion, plasmapheresis or even emergency splenectomy as initial treatment ( [5,6]). Initial response to conventional prednisolone therapy (1-2 ^mg^/_kg_/day) varies from 65% to 85%, but sustained response has been reported to be less than 25% ( [7]). Terminology used in this manuscript was according to an international working group in 2009 ( [8]). Use of high dose dexamethasone as initial treatment in ITP is controversial, however a number of studies have reported good response rate with minimal toxicity ( [9-11]). The aim of this perspective randomized trial was to evaluate effectiveness of single course of high dose dexamethasone comparing conventional prednisolone therapy in the treatment of newly diagnosed adult ITP patients.

## Methods

In a single-blind randomized prospective study in Ali-Ebne-Abitaleb hospital in Zahedan, newly diagnosed adult patients with ITP were evaluated. Our inclusion criteria were: newly diagnosed primary ITP with platelet count less than 20,000/microliter. Bleeding tendency was graded as a previously definition (Table 1) ( [12]). The study protocol was approved by regional medical ethical committee **(Approved No; 328, 2007.2.24**) and all of the patients provided informed consent. Patients with previously treated ITP, diabetes, liver and kidney dysfunction, infections including HIV, HCV, and HBV, pregnancy and SLE or any acute illness were excluded. ITP was diagnosed according to clinical features, CBC, and bone marrow aspiration ( [13,14]). After initial clinical evaluation and performing CBC, bone marrow aspiration was done. Collagen vascular diseases or anti phospholipid antibody and other hematologic disorders were ruled out using appropriate tests. Patients randomly divided into two groups: group A received dexamethasone 40 mg/day/IV (10 mg/q6h) for four days and then prednisolone 1^mg^/_kg_/day/PO with 10 mg/week tapering and discontinuation after 6 weeks. Group B treated with prednisolone 1^mg^/_kg_/day for four weeks and then tapering 10 mg/week. Peripheral blood smear and CBC checked daily during hospitalization, then weekly for 6 weeks, and then monthly. Response to treatment was classified to complete response (CR) (PLT count >100,000), response (R) (PLT count ≥30,000) and no response (NR) (no rising in PLT or PLT count < 30,000 or symptomatic bleeding) [8]. Patients were followed for at least 12 months (ranged 12-48 months) (Table 2, 3). Toxicities were graded according to CTCAE criteria ( [12,15]).

**Table 1:** Grade and type of bleeding symptom

**Grade**	**Type of bleeding**
0	Absent
1	Petechiae
2	Ecchymoses and/or dripping with moderate loss of blood
3	Major mucous hemorrhage with copious loss of blood without sequelae
4	Major mucous and/or parenchymal hemorrhage with copious loss of blood with sequelae and/or life-threatening

**Table 2:** General characteristics of patients

	**Group A**	**Group B**	**P value**
Sex(F/M)	24/6	23/7	0.75
Mean age(Year)	24.9 (17.5 – 44)	27.2 (18 – 48.4)	0.49
Mean platelet count (μL)	13900 (3400 – 18000)	10400 (1500 – 16400)	0.56
Grade of bleeding (No.)			
0	0	0	
1	16	19	
2	8	7	
3	6	4	
4	0	0	

**Table 3:** Comparison of outcomes in group A versus group B

		**Group A No. (%)**	**Group B No. (%)**	**P value**
One week				0.024
	CR	27 (90)	11 (36.7)	
	R	3 (10)	13 (43.3)	
	NR	0 (0)	6 (20)	
Three months				< 0.0001
	CR	24 (80)	7 (23.3)	
	R	5 (16.7)	10 (33.3)	
	NR	2 (6.6)	13 (43.3)	
Six months				< 0.0001
	CR	22 (73.3)	5 (16.6)	
	R	5 (16.6)	11 (36.7.3)	
	NR	3 (10)	14 (46.7)	
One year				< 0.0001
	CR	22 (73.3)	2 (6.6)	
	R	5 (16.6)	12 (40)	
	NR	3 (10)	16 (53.3)	
Splenectomy		3 (10)	12 (40)	< 0.0002

Statistical analysis: Data analysis was performed using SPSS version 16 (SPSS Inc, Chicago, IL, USA). Independent *T* test was used for comparison of continuous variables. Differences in the distribution of variables between groups were analyzed by the *X*^2^ or Fisher exact test. P < 0.05 was regarded as significant.

## Results

Sixty patients completed the study and came for weekly follow up visits and laboratory evaluations. These consecutive patients randomly assigned to group A and B. There was no significant difference between mean age (P = 0.49), sex (P = 0.75) and platelet count (P = 0.56) of the two groups (Table 2).

Seven days after initiation of treatment protocol, in group A, CR and R were observed in 27 and three patients, respectively. In group B, we showed CR in 11 cases, R in 13 cases and NR in six cases. After three months, in group A, two patients were NR, five cases showed R and 23 cases revealed CR and loss of CR and R occurred in this period. In group B, NR, R and CR were observed in 13, 10 and seven patients, respectively (P < 0.0001). After 6 months, in group A versus group B, the number of patients with NR, R and CR were three versus 14, five versus 11 and 22 versus five (P < 0.0001). One year after the initial treatment, there were no further changes in group A; however, in group B, number of NR cases increased to 16, two cases showed CR and the remaining cases were in R group (P < 0.0001). Splenectomy as a second line treatment was performed for three cases in A group and for 12 cases in group B (P < 0.00002) (Table 3).

Overall, treatment related toxicities (Table 4) were minimal. Adverse reactions in group B were more common than group A. In group A, treatment related toxicities were weight gain in five cases (17.3%) (All grade 1), abnormal glucose level (grade 2 toxicity) needed transient treatment in one case, gastrointestinal distress in two cases (one case in grade 2 and another in grade three toxicity) and grade one hypertension in one case. In group B, the most common toxicities were weight gain in 13 cases (43.3%) with grade one toxicity, hypertension in three cases (10%), (one case in grade 2 and two cases in grade 1 toxicity), abnormal glucose level needed transient treatment in five cases (16.5%) (three in grade 2 and two in grade one of toxicity), and gastrointestinal toxicity in seven (23.3%) patients, (five in grade 2 and two in grade 3 of toxicity).

**Table 4:** Treatment related toxicity

	**Group A No. (%)**	**Group B No. (%)**
Weight gain	5(17.3)	13(43.3)
	Grade of toxicity : 1	Grade of toxicity : 1
Glucose intolerance	1(3.3)	5(16.5)
	Grade of toxicity : 2	Grade of toxicity :
		3 cases grade : 2
		2 cases grade : 1
Hypertension	1(3.3)	3(10)
	Grade of toxicity : 1	Grade of toxicity :
		One case grade : 2
		2 cases grade 1
Gastrointestinal distress	2(6.6)	7(23.3)
	Grade of toxicity : 1	Grade of toxicity : 1
	1 case grade 2	5 cases: grade 2
	1 case grade 3	2 cases : grade 3

## Discussion

The standard treatment of ITP is corticosteroid therapy, mainly prednisolone at 1^mg^/_kg_ for four weeks and then tapering ( [16]). Although response rate to this therapeutic regimen has been reported 50-60%, durability is low (10-25%) ( [7,17]).Our results showed that single course of high dose dexamethasone regimen is more effective than conventional therapy as an initial treatment for ITP patients.

Cheng and colleagues demonstrated that 85% of all cases received high dose dexamethasone had initial response, while half of them had sustained response. Other cases experienced relapse disease (PLT < 30,000). However, 50% of these cases only needed 10 mg/day prednisolone to continue response after second course of high dose dexamethasone and other cases needed more than 10 mg/day plus further treatment including splenectomy, IVIG, Anti D, danazol, vina alkaloid, and Cyclosporine. In our study initial response was similar to Cheng study, but we showed higher sustained and durable response and lower relapse rate (16.6% vs. 50%). This difference may be due to younger age of our patients and continuation of prednisolone for at least four weeks. The later probable reason may be supported by this fact that in cheng study 50% of relapses responded to 10 mg prednisolone ( [9]).

In another study Borst and coworkers treated 36 cases with high dose dexamethasone; 50% as first line therapy and 50% as second line. In first line group 59% had durable response (CR + PR) for 2-31 months, 28% had transient response with immediate relapse after cessation of high dose dexamethasone and 11% had no response ( [18]). In second line group 25% had durable response (CR + PR) for 4 – 54 months, 61% had transient response and 11% had no response. In both groups 30.5% of patients underwent splenectomy. In this study 44% had no evidence of any side effect, 13.8% had severe toxicity and refused this modality of treatment.

In 2007 Mazzucconi and colleagues conducted two studies: In a mono-center study 37 adult patients treated with 4-6 cycles of high dose dexamethasone every 28 days. Response rate was 89.2% (CR :62.2%, PR: 21.6%, MR: 5.4%, and NR in 10.8%). In this study, relapse rate and durable response were 24.2% and 75%, respectively. Sustained response was similar to our study (67.6% vs. 75%). Thirty three cases showed a favorable response to dexamethasone therapy and relapse occurred in eight cases which were higher than our ones. In the Multicenter study response rate was 85.6% (CR 64.5%; PR 20%; MR 1.1% and NR14.4%). With administration of every other course of high dose dexamethasone therapy, response rate increased (69.5%, 75.5%, 89%). The quality of response in younger patients was more favorable than older patients (P = 0.039) and relapse rate was lower (P = 0.01). Age was the most important factor for response and relapse in Mazzucconi study. After 6.5 months of treatment, relapse rate was 13% among responders. Durable response without any treatment after 8 months follow up was 74.4% ( [13]).

Compared to multicenter results, our patients were adults and duration of follow up was longer (12-48 vs. 3-24 months). Durable response was similar in both studies; however CR was lower in multicenter study (64.5% vs. 80%) and relapse rate was higher (22% vs. 16.6%).

The younger mean age of our patients and continuation of glucocorticoid after cessation of dexamethasone, may explain more favorable response especially higher durable response in our study.

The favorable and better outcome of current study comparing Mazzucconi and Cheng studies were: young age of our patients and continuation of corticosteroid administration after cessation of high dose dexamehasone. Continuation of prednisolone may be effective in sustained blockade of clearance system and inhibition of antibody synthesis and deposition ( [19-22]).

The strengths of our study were: the first randomized prospective study about comparison between high dose dexamethasone and conventional prednisolone therapy, being all cases in severe disease according to PLT count and doing close follow up visits. Our limitations were: Age distribution (excluding childhood and older cases), and single-blind study (instead of double-blind), and limited number of patients (only 30 cases in every groups).

## Conclusion

Comparing conventional treatment, our study showed more effectiveness of single course of high dose dexamethasone with continuation and rapid tapering of prednisolone as an initial treatment for primary newly diagnosed immune thrombocytopenia with minimal and acceptable side effects. We recommend further head to head studies to evaluate effectiveness of this regimen comparing repeated cycles of dexamethasone as sole treatment.

## Competing interest

The authors declare that they have no competing interest.

## Author's contribution

All of the authors had the same contribution in article.

## References

[B1] Chong BH Keng TB Advances in the diagnosis of idiopathic thrombocytopenic purpura Semin Hematol 2000 3 249 260 10942219 10.1016/s0037-1963(00)90103-3

[B2] Cines DB Blanchette VS Immune thrombocytopenic purpura N Engl J Med 2002 346 995 1008 10.1056/NEJMra010501 11919310

[B3] George JN el-Harake MA Raskob GE Chronic idiopathic thrombocytopenic purpura N Engl J Med 1994 331 1207 1211 10.1056/NEJM199411033311807 7935660

[B4] Douglas B Cines M Victor S Blanchett MB Immune thrombocytopenic pupura N Engl J Med 2002 346 995 1007 10.1056/NEJMra010501 11919310

[B5] Cines DB McMillan R Management of adult idiopathic thrombocytopenic purpura Annu Rev Med 2005 56 425 442 10.1146/annurev.med.56.082103.104644 15660520

[B6] Newland A Caulier MT Kappers-Klunne M Schipperus MR Lefrere F Zwaginga JJ Christal J Chen CF Nichol JL An open-label, unit dose-finding study of AMG 531, a novel thrombopoiesis-stimulating peptibody, in patients with immune thrombocytopenic purpura British Journal of Hematology 2006 135 547 553 10.1111/j.1365-2141.2006.06339.x 17061981

[B7] Gupta V Tilak V Bhatia BD Immune thrombocytopenic purpura Indian J Pediatr 2008 75 723 728 10.1007/s12098-008-0137-z 18716743

[B8] Rodeghiero F Stasi R Gernsheimer T Michel M Provan D Arnold DM Bussel JB Cines DB Chong BH Cooper N Standardization of terminology, definitions and outcome criteria in immune thrombocytopenic purpura of adults and children: report from an international working group Blood 2009 113 2386 2393 10.1182/blood-2008-07-162503 19005182

[B9] Cheng Y Wong RS Soo YO Chui CH Lau FY Chan NP Initial treatment of immune thrombocytopenic purpura with high-dose dexamethasone N Engl J Med 2003 349 831 836 10.1056/NEJMoa030254 12944568

[B10] Andersen JC Response of resistant idiopathic thrombocytopenic purpura to pulsed high-dose dexamethasone therapy N Engl J Med 1994 330 1560 1564 10.1056/NEJM199406023302203 8177245

[B11] Khouri I Tuan B Grant K Immune thrombocytopenic purpura N Engl J Med 2002 347 449 450 12167694 10.1056/NEJM200208083470617

[B12] National Cancer Institute. DCTD, NCI, NIH and DHHS. Cancer therapy. Evaluation program, Common terminology Criteria for adverse event Version 3.0. http://Ctep.Cancer.gov.2.2003.

[B13] Mazzucconi MG Fazi P Bernasconi S De Rossi G Leone G Gugliotta L Vianelli N Avvisati G Rodeghiero F Amendola A Therapy with high-dose dexamethasone (HD-DXM) in previously untreated patients affected by idiopathic thrombocytopenic purpura: a GIMEMA experience Blood 2007 109 1401 1407 10.1182/blood-2005-12-015222 17077333

[B14] George JN Woolf SH Raskob GE Wasser JS Aledort LM Ballem PJ Idiopathic thrombocytopenic purpura: a practice guideline developed by explicit methods for the American Society of Hematology. Blood, 1996;88:3-40. 15. Rahimi R, Nikfar S, Abdollahi M. Meta-analysis finds use of inhaled corticosteroids during pregnancy safe: a systematic meta-analysis review Hum Exp Toxicol 2006 25 447 452 10.1191/0960327106het647oa 16937916

[B15] Shad AT Gonzalez CE Sandler SG Treatment of immune thrombocytopenic purpura in children: current concepts Paediatr Drugs 2005 7 325 336 10.2165/00148581-200507050-00004 16220997

[B16] Bellucci S Charpak Y Chastang C Tobelem G Low doses v conventional doses of corticoids in immune thrombocytopenic purpura (ITP): results of a randomized clinical trial in 160 children, 223 adults Blood 1988 71 1165 1169 2451549

[B17] Borst F Keuning JJ van Hulsteijn H Sinnige H Vreugdenhil G High-dose dexamethasone as a first- and second-line treatment of idiopathic thrombocytopenic purpura in adults Ann Hematol 2004 83 764 768 10.1007/s00277-004-0908-1 15309522

[B18] Liu XG Ma SH Sun JZ Ren J Shi Y Sun L High-dose dexamethasone shifts the balance of stimulatory and inhibitory Fcgamma receptors on monocytes in patients with primary immune thrombocytopenia Blood 2011 117 2061 2069 10.1182/blood-2010-07-295477 21131591

[B19] Zhu XJ Shi Y Sun JZ Shan NN Peng J Guo CS Qin P Hou M High-dose dexamethasone inhibits BAFF expression in patients with immune thrombocytopenia J Clin Immunol 2009 29 603 610 10.1007/s10875-009-9303-y 19499314

[B20] Shan NN Zhu XJ Wang Q Wang CY Qin P Peng J High-dose dexamethasone regulates interleukin-18 and interleukin-18 binding protein in idiopathic thrombocytopenic purpura Haematologica 2009 94 1603 1607 10.3324/haematol.2009.007708 19797730 PMC2770973

[B21] Fujisawa K Tani P Piro L McMillan R The effect of therapy on platelet-associated autoantibody in chronic immune thrombocytopenic purpura Blood 1993 81 2872 2877 8499625

[B22] Hsu NC Chung CY Horng HC Chang CS Corticosteroid administration depresses circulating dendritic cells in ITP patients Platelets 2004 15 451 454 10.1080/09537100410001711497 15745317

